# Quantitative detection of *DNMT3A* R882H mutation in acute myeloid leukemia

**DOI:** 10.1186/s13046-015-0173-2

**Published:** 2015-05-22

**Authors:** Rimma Berenstein, Igor Wolfgang Blau, Nikola Suckert, Claudia Baldus, Antonio Pezzutto, Bernd Dörken, Olga Blau

**Affiliations:** Department of Hematology, Oncology and Tumorimmunology, Charité University School of Medicine, Hindenburgdamm 30, 12200 Berlin, Germany

**Keywords:** AML, *DNMT3A* mutations, Quantitative PCR

## Abstract

**Background:**

*DNMT3A* mutations represent one of the most frequent gene alterations detectable in acute myeloid leukemia (AML) with normal karyotype. Although various recurrent somatic mutations of *DNMT3A* have been described, the most common mutation is located at R882 in the methyltransferase domain of the gene. Because of their prognostic significance and high stability during disease evolution, *DNMT3A* mutations might represent highly informative biomarkers for prognosis and outcome of disease.

**Methods:**

We describe an allele-specific PCR with a Blocking reagent for the quantitative detection of *DNMT3A* R882H mutation providing the possibility to analyze the quantitative amount of mutation during the course of disease. Next, we analyzed 62 follow-up samples from 6 AML patients after therapy and allogeneic stem cell transplantation (alloSCT).

**Results:**

We developed an ASB-PCR assay for quantitative analysis of R882H *DNMT3A* mutation. After optimization of blocker concentration, a R882H-positive plasmid was constructed to enhance the accuracy of the sensitivity of quantitative detection. The assay displayed a high efficiency and sensitivity up to 10^−3^. The reproducibility of assay analyzed using follow-up samples showed the standard deviation less than 3.1 %. This assay displayed a complete concordance with sequencing and endonuclease restriction analysis. We have found persistence of *DNMT3A* R882H mutations in complete remission (CR) after standard cytoreduction therapy that could be indicating presence of *DNMT3A* mutation in early pre-leukemic stem cells that resist chemotherapy. The loss of correlation between *NPM1* and *DNMT3A* in CR could be associated with evolution of pre-leukemic and leukemic clones. In patients with CR with complete donor chimerism after alloSCT, we have found no DNMT3A R882H. In relapsed patients, all samples showed an increasing of both *NPM1* and *DNMT3A* mutated alleles. This suggests at least in part the presence of *NPM1* and *DNMT3A* mutations in the same cell clone.

**Conclusion:**

We developed a rapid and reliable method for quantitative detection of *DNMT3A* R882H mutations in AML patients. Quantitative detection of *DNMT3A* R882H mutations at different time points of AML disease enables screening of follow-up samples. This could provide additional information about the role of *DNMT3A* mutations in development and progression of AML.

## Introduction

Somatic mutations in the DNA nucleotide methyltransferase 3A gene (*DNMT3A)* have been reported approximately in 22 % of *de novo* acute myeloid leukemia (AML) and 36 % of cytogenetically normal AML [1]. Mutations in *DNMT3A* were first described by Ley *et al.* using whole genome sequencing [2]. *DNMT3A* belongs to the mammalian methyltransferase gene family which is responsible for tissue-specific gene expression [3]. DNA methyltransferases are the key enzymes for genome methylation, which plays an important role in epigenetically regulated gene expression and repression. *DNMT3A* together with other methyltransferases conducts *de novo* methylation of cytosine residues in CpG islands by the enzymatic addition of methyl residues from S-adenosyl-L-methionine to the 5-carbon atom of the cytosine ring. CpG islands are often located proximate to gene promoters thereby regulating their activation. Actively transcribed genes display unmethylated CpG islands which supports the euchromatin structure whereas methylated CpG islands are associated with untranscribed genes stabilizing the heterochromatin structure [4, 5]. Cancer genomes are most commonly characterized by global DNA hypomethylation. However, cancer cells also typically exhibit distinct regions of DNA hypermethylation, which are particularly well characterized in the CpG islands of promoter regions of tumor-suppressor genes. Although various recurrent *DNMT3A* mutations have been described, the most common mutation affects residue R882 within the methyltransferase domain. *DNMT3A* mutations are typically heterozygous [2, 6].

The biology of *DNMT3A* is not fully understood. Holz-Schietinger *et al.* reported that mutations in *DNMT3A* could retard its function by multiple mechanisms as changes in the catalytic properties, its processivity and the disruption of interaction with binding partners [7]. Furthermore, Russler-Germain *et al.* found that mutations in the position R882 inhibit the formation of active tetramers of *DNMT3A* [8]. The impaired function of mutated *DNMT3A* leads to a hypomethylated genome of myeloid cells possibly promoting leukemogenesis and influencing disease outcome [9].

Since the *DNMT3A* mutations are present in the early pre-leukemic cells, this alteration seems to be a “founder” mutation, which can be implicated as functional components of AML evolution [10, 11]. *DNMT3A* mutations are highly associated with mutations in the nucleophosmin 1 gene (*NPM1*), fms-related tyrosine kinase 3 gene (*FLT3*), and isocitrate dehydrogenase 1 gene (*IDH1*) [12, 13].

Several studies reported a negative prognostic impact of *DNMT3A* mutations [12–16]. Prognostic effect is known to depend on certain biological factors as well as a combination of cytogenetics and other mutations such as those in *FLT3* and *NPM1*.

Some authors have found stability of *DNMT3A* mutations during the course of disease; therefore those aberrations could be potential marker for minimal residual disease (MRD). Furthermore, the presence of *DNMT3A* mutations seems to be associated with the incidence of *FLT3-*ITD-positive clones at relapse possibly influencing the responsiveness of *FLT3*-positive cases to chemotherapy [17, 18]. Last published data have demonstrated that *DNMT3A* mutations are also detectable in AML patients in long-term complete remission (CR) and can occur in pre-leukemic stem cells [10, 11, 19]. The identification of pre-leukemic cells with genetic mutations in CR has important implications for the MRD monitoring. Moreover, the persistence of *DNMT3A* mutations in CR may have important implications for the management of AML.

Recent discoveries utilizing whole-exome sequencing in a large cohort of persons, unselected for cancer or hematologic phenotypes have demonstrated somatic mutations in significant proportion of persons particularly which older than 65 years. Moreover, *DNMT3A* gene together with *TET2, ASXL1,* and *PPM1D* had disproportionately high numbers of somatic mutations [20, 21]. The data suggest that mutations in pre-leukemic cells could precede leukemia. Furthermore, *DNMT3A* mutations could drive clonal expansions. Based on these data, *DNMT3A* mutation might represent highly informative biomarkers for AML. Thus, a negative prognostic impact and, in addition, conflicting reports on the potential role of *DNMT3A* mutations for the evolution of leukemic stem cell, require fast, reliable, quantitative and available methods for detection of mutation.

Sanger sequencing is well-established but not very sensitive as well as time-consuming and cost-intensive method. HRM analysis provides the possibility of high throughput screening of mutations, but data interpretation occasionally can be difficult. Therefore, good validated controls and standards are needed. Previously we reported a rapid and reliable restriction fragment length polymorphism based method for the qualitative detection of *DNMT3A* R882H mutation [22]. Current quantitative established assays have low sensitivity and therefore cannot be used as reliable methods for MRD diagnostic. Here, we describe an Allele-Specific PCR with a Blocking reagent (ASB-PCR) for the quantitative detection of *DNMT3A* R882H mutation with sensitivity up to 10^−3^ providing the possibility to analyze the quantitative amount of this mutation for routine diagnostic during the course of disease.

## Methods

### Patient and control materials

Bone marrow (BM) samples from 16 newly diagnosed AML patients, 8 *DNMT3A* positive and 8 *DNMT3A* wild type (wt), were included in the study. In addition, we analyzed 62 follow-up samples from 6 *DNMT3A* positive patients after chemotherapy and allogeneic stem cell transplantation (alloSCT). All patients were treated at the University Clinic Charité from September 2009 to May 2013. Diagnoses were established according to the WHO criteria [23]. Written informed consent was obtained from all patients in accordance with the Declaration of Helsinki and the ethical guidelines of the Charite University School of Medicine, which approved this study. In 6 AML patients included in the follow up study, induction therapy consisted of “7 + 3” therapy with Cytosine Arabinoside and Daunorubicine. Second part of induction with “7 + 3” started at day 22, if at day 15 count of BM blasts were reduced. Once complete remission (CR) was achieved, 2–4 courses of consolidation chemotherapy with high-dose Cytosine Arabinoside were administered. At 1-st CR or at 2-nd CR patients were allocated to alloSCT.

Control material for *DNMT3A* mutation included DNA from K562 cell line (wt) and constructed plasmid which contains the R882H mutation.

### DNA extraction

Mononuclear cells (MNCs) from BM aspirates were isolated using Ficoll density centrifugation as described [24]. DNA was extracted using Allprep DNA/RNA mini kit (Qiagen) from 1*10^7^ MNCs as recommended by the manufacturer. DNA yields ranged from 50 to 300 ng/μl.

### Allele-Specific PCR with a Blocking reagent (ASB-PCR)

**Fig. 1:**
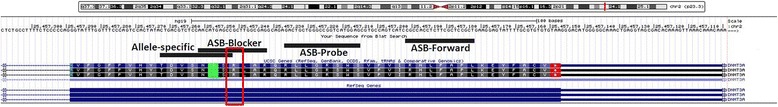
Assay design of ASB-PCR. Primers and probe are located in exon 23 of *DNMT3A*. The allele-specific primer contains the mutational spot at its 3′-end whereas the wt spot is incorporated in the middle of the blocker (red box). Fluorescence detection was performed with a TaqMan probe which was designed near to the forward primer (13 bp distance)

The ASB-PCR assay was designed using Primer3, Oligocalc and UCSC software. We used an allele-specific reverse primer containing the mutational spot (R882H G > A) at its 3′-end. The blocking sequence was developed complementary to the wt allele. The discriminating base was located in the middle of the blocker. To prevent elongation by Taq polymerase, a phosphate group was added to the 3′-end of the blocker. For fluorescence detection, a TaqMan probe was used (Fig. 1). All primer sequences are listed in Table 1. Further properties, such as melting temperature were developed in accordance with Morlan *et al.* [25]. The reaction mixture contained 12.5 μl 2 × Absolute qPCR Mix (Applied Biosystems), 10 pmol of each forward and reverse allele-specific primer, 40 pmol of ASB-Blocker, 5 pmol of probe, and 30 ng of DNA in a final reaction volume of 25 μl. Reaction was run at 95 °C for 10 min followed by 40 cycles of denaturation at 95 °C for 20 s and annealing/elongation at 67 °C for 45 s on a Rotor Gene 6000 Real-Time PCR Cycler (Qiagen).

**Table 1:** Oligonucleotides used in this study

Name	Sequence	Application	Tm [°C]
*DNTM3A*-Ex23F	5′-GTGTGGTTAGACGGCTTCC	Sequencing	59.5
*DNMT3A*-Ex23R	5′-CTCTCCCACCTTTCCTCTG	Sequencing	59.5
ASB-F	5′- CAGCGGAGCGAAGAGGTG	ASB-PCR	60.8
Allele-specific	5′- CGTCTCCAACATGAGCC***A***	ASB-PCR	56.3
ASB-Blocker	5′- CATGAGCC***G***CTTGGCGAG-PH	ASB-PCR	60.8
ASB-Probe	5′- FAM-CTCCATGACCGGCCCAGCAGTC-BBQ	ASB-PCR	69.5

The mutational spot in the allele-specific primer and blocker sequence is bold and punctuated. ASB, indicates Allele-Specific PCR with a Blocking reagent; *DNMT3A*, indicates DNA methyltransferase 3 gene

### Plasmid preparation

For absolute quantification, a plasmid containing the *DNMT3A* R882H G > A mutations was constructed. The template was amplified using ASB-PCR primers, and the fragment size (94 bp) was analyzed on a 1.5 % agarose gel. The plasmid was prepared using the TOPO TA cloning Kit (Invitrogen) and chemically competent *E.coli* as per the manufacturer’s instructions. Fragment insertion was checked using DNA sequencing with provided M13 forward and reverse primers.

### DNA sequencing and endonuclease restriction analysis of *DNMT3A* mutations

PCR sequencing reaction was performed as previously described [22]. Amplified products were purified using the PCR Purification Kit (Qiagen) according to the manufactures instruction. Sequencing was performed using ABI310 Genetic Analyzer (Applied Biosystems), and data were analyzed using DNA Sequencing Analysis Software v.5.2.0. Endonuclease restriction analysis of *DNMT3A* R882H mutation was performed using Fnu4HI (New England Biolabs) as previously reported [22].

### Qualitative and quantitative evaluation of mutations

Qualitative evaluation for presence of *NPM1*, *DNMT3A*, *IDH1* and *IDH2* mutations were performed by Sanger sequencing using ABI310 Genetic Analyzer (Applied Biosystems) as previously described [22]. *FLT3*-ITD and *FLT3* D835Y were quantified by fluorescence fragment analysis on a 310 Genetic Analyzer (Applied Biosystems) as previously described [26]. Quantitative analysis of presence *NPM1* mutations was performed by real-time PCR using Rotor Gene 6000 Real-Time PCR Cycler (Qiagen). The reaction mixture contained 12.5 μl 2 × Absolute qPCR Mix (Applied Biosystems), 30 pmol of each forward and reverse allele-specific primer, 10 pmol of forward and 6 pmol of reverse HCK probes, 25 pmol of each FAM-MGB and HEX probes, and 50 ng of DNA in a reaction final volume of 25 μl. Reaction was run at 95 °C for 15 min followed by 45 cycles of denaturation at 95 °C for 15 s and annealing/elongation at 60 °C for 1 min.

## Results

### ASB-PCR assay performance

**Fig. 2:**
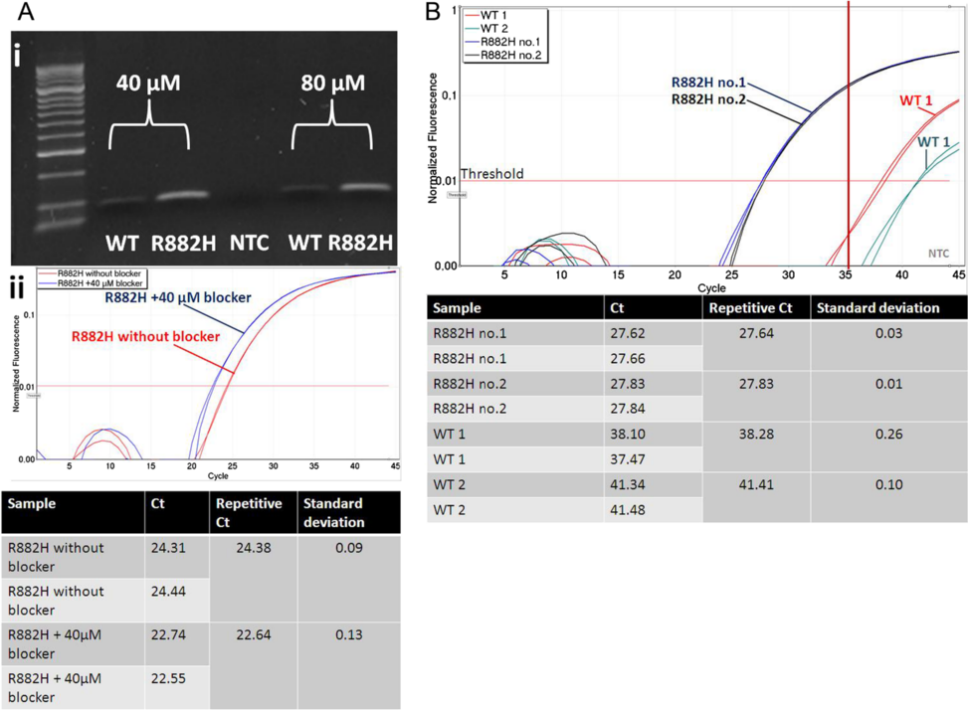
Performance of ASB-PCR. **ai** Qualitative analysis of the specificity of ASB-PCR. Enhancement of blocker concentration to 80 μM showed no significant change in the amplification properties. **aii** Quantitative analysis of Ct-changes induced by addition of 40 μM blocker. **b** Quantitative analysis displayed a C_t_ difference of 11–14 cycles between R882H mutated DNA and wt DNA. NTC, non-template control; wt, wild type

At first, we have tested the performance of ASB-PCR by standard PCR amplification and agarose gel electrophoresis. Specificity of ASB-PCR was verified using Sanger sequencing. To determine the optimal blocker concentration, PCR was performed with 40 μM and 80 μM of blocker, successively. A specific amplification of mutated DNA was determined producing a PCR fragment of approximately 94 bp. DNA of the wt sample was also amplified, but with a considerably lower efficiency compared to R882H-positive DNA (Fig. 2.ai). No significant difference was seen for PCR amplification with 40 μM and 80 μM of blocker. However, the presence of 40 μM blocker in the reaction led to detection of R882H around 2 Ct’s earlier compared to the assay without blocker (22.64 *vs.* 24.38; Fig. 2.aii). Therefore, 40 μM of blocker were used for further applications. Next, we determined the quantitative performance of the assay (Fig. 2.b). The fluorescence signal of mutated samples exceeded the threshold 11–14 cycles earlier as compared with wt samples (27.64/27.83 *vs.* 38.28/41.41). Based on this analysis, we chose a C_t_-value of 35 as the cut-off limit.

**Fig. 3:**
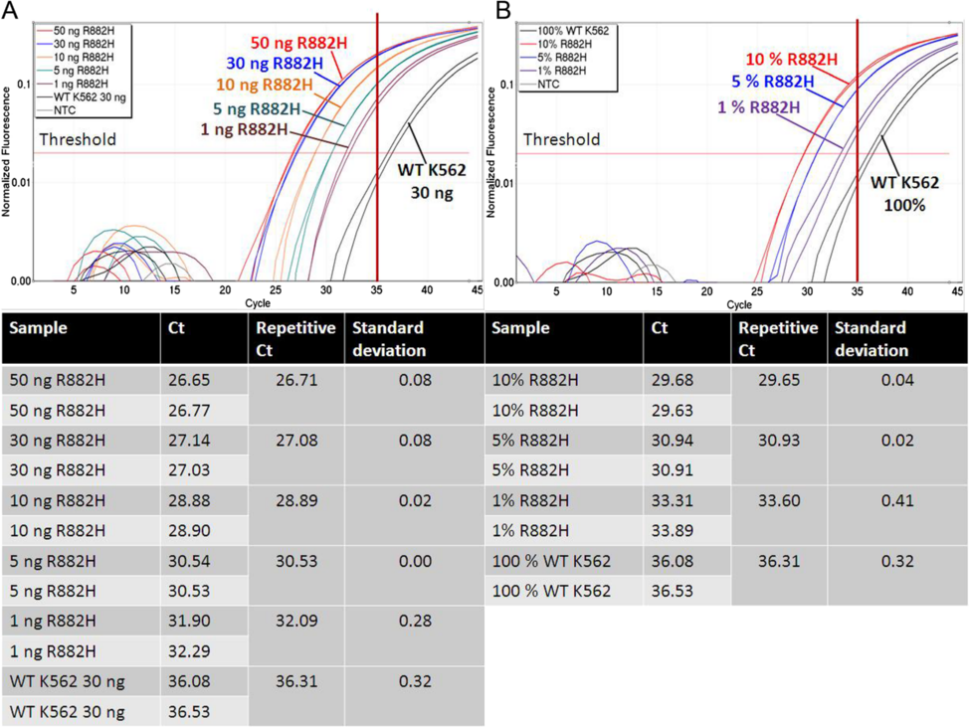
Analysis of PCR sensitivity using serial dilutions of DNA (**a**) and (**b**) dilution of mutated DNA with wt DNA

Subsequently, the sensitivity of ASB-PCR was studied. First, a dilution series of genomic DNA was prepared ranging from 50 ng to 1 ng (Fig. 3.a). Differentiation between wt and R882H samples was possible for all dilution steps. One ng of R882H DNA was detected 4 cycles earlier compared with 30 ng of wt DNA (32.09 *vs.* 36.31). In addition, a dilution series of R882H-positive DNA with wt DNA was analyzed (Fig. 3.b). Discrimination of 1 % of R882H-positive DNA was possible exceeding the threshold approximately 3 cycles earlier as the wt DNA (33.60 *vs.* 36.31). The cut-off limit of C_t_ = 35 was constant for these analyses. To enhance the accuracy of the sensitivity determination of quantitative detection of the mutation, a R882H-positive plasmid was constructed.

### Plasmid performance and absolute quantification

**Fig. 4:**
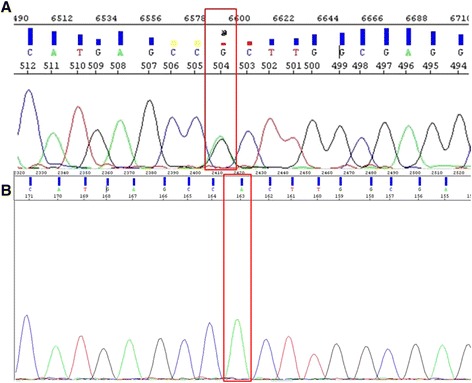
Sequence of R882H plasmid. **a** Heterozygote R882H mutation in a patient sample. The mutational spot is indicated by the red box. Both, guanine (wt) and adenine (mutation) were detected. **b** Homozygote R882H mutation in the generated plasmid. The mutational spot is indicated by the red box. The sequence displays only the mutated adenine base and no wt guanine base

**Fig. 5:**
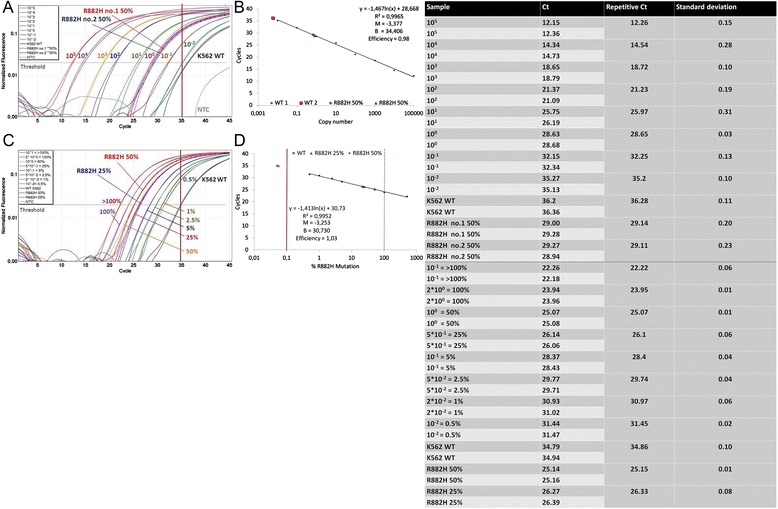
Absolute quantification of *DNMT3A* R882H mutations. Values of ASB-PCR are listed in the corresponding table. **a** Analysis of copy number dilutions of the R882H homozygote plasmid. Patient samples with approximately 50 % of R882H mutated allele are indicated by dotted lines; the 10° plasmid dilutions are pink line. **b** The standard curve (copy numbers over cycle). Patient samples with 50 % R882H are shown as a blue diamond and a green triangle. Wt samples are shown as red quadrats. R^2^ = correlation coefficient. B = Intercept with the ordinate. M = Slope of the standard curve. **c** Absolute quantification was performed by percentage of mutated allele in a sample. Plasmid concentrations were adjusted to 50 % and 25 % of mutated R882H allele according to the measurement of heterozygote patient samples (dotted lines). **d** Standard curve (%R882H over cycle). The dotted red line shows the limit of 100 % R882H mutated allele in a sample. Patient samples are shown as a blue diamond and a green triangle. wt samples are shown as red quadrats. R^2^ = correlation coefficienct. B = Intercept with the ordinate. M = Slope of the standard curve

We have generated a plasmid which contains a homozygous R882H mutation (Fig. 4). Significant differentiation to the wt DNA was possible up to a dilution of 10^−1^ copies. A copy number of 10^−2^ exceeded the threshold approximately at the cut-off value of 35 cycles (Fig. 5.a). However, the assay effectiveness was distinct when the plasmid containing a homozygous R882H mutation was compared with heterozygous patient samples. Two samples obtained from AML patients at diagnosis containing approximately 50 % of *DNMT3A* R882H mutations exceeded the threshold at a similar cycle as the 10° dilution of the plasmid (29.14/29.11 *vs.* 28.65; Fig. 5.b). The initial mutation portion was determined by Sanger sequencing. Since the absolute quantification was not possible in this setting, we performed an absolute quantification by the percentage of mutated allele. Based on the previous measurement, the 10° plasmid dilution was defined to contain 50 % of *DNMT3A* R882H mutation. Fig. 5.c demonstrates a possibility of reliable quantification of patient samples using this setting. The sample containing 50 % of *DNMT3A* R882H was detected at the same cycle as the 10^0^ plasmid dilution (25.15 *vs.* 25.07). In addition, the sample with 25 % R882H mutation exceeded the threshold at a similar cycle compared to the 5*10^−1^ plasmid controls (26.33 *vs.* 26.1). The assay displayed a high efficiency between 0.98 and 1.03 (Fig. 5.b and d). The sensitivity of assay ranged up to 10^−3^.

In addition, follow-up samples of one patient were analyzed three times to examine the reproducibility of assay results. As shown in Table 2 the standard deviation was ≤ 3.1 %.

**Table 2:** Reproducibility of ASB-PCR

Sample	Run 1 (%R882H)	Run 2 (%R882H)	Run 3 (%R882H)	Standard deviation (%)
187	23.05	21.6	17.19	3.052
187-1	8.2	8.65	8.4	0.225
187-2	3.5	3.5	2.82	0.392
187-3	2.1	1.4	1.07	0.525
187-4	2.35	3.35	1.70	0.831
187-5	31.35	33.05	35.3	1.981

### Assays concordance

Next, the concordance of different methods for detection of *DNMT3A* mutation was examined.

**Fig. 6:**
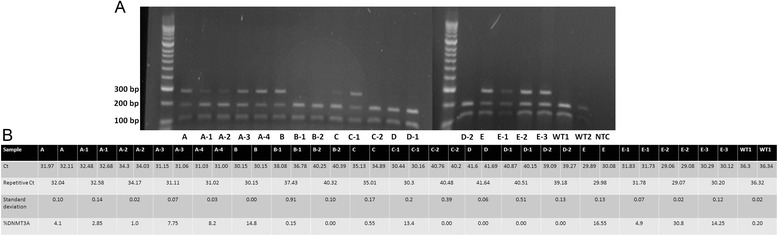
Concordance of ASB-PCR. **a** Representative endonuclease restriction analysis of follow-up samples of five patients (A-E). Wild type samples show two bands at 190 bp and 114 bp. Positive samples display three bands at 289 bp, 190 bp, 114 bp due to the loss of a restriction site of Fnu4HI caused by the mutation. Hyperladder II (Bioline) was used as marker. **b** Results of ASB-PCR analysis

Initially, 25 samples from AML patients were analyzed using DNA sequencing. Next, endonuclease restriction analysis and ASB-PCR were used (Fig. 6). We have previously described that endonuclease restriction analysis has a perfect concordance with Sanger sequencing and is characterized by high sensitivity [22]. Qualitative assessment of band sizes (Fig. 6.a) corresponded to quantitative detection of R882H mutation using ASB-PCR (Fig. 6.b) in follow up samples from patient A, B, C, und E. Patient D showed no *DNMT3A* R882H mutation using both methods. For example, increasing of *DNMT3A* R882H mutation in samples from patient C from 0.55 % to 13.4 % by ASB-PCR assay (Fig. 6.b) matched with a gain of band by endonuclease restriction analysis (Fig. 6.a). A cut-off of Ct = 35 was also applicable for analysis of follow-up samples. No false negative or positive signals were detected using the endonuclease restriction and ASB-PCR.

### Analysis of follow-up samples from AML patients

We analyzed 62 follow up constitutive samples obtained from 6 AML patients at diagnosis, after induction, consolidation therapy, and after alloSCT. All patients included in the follow-up analysis harbored a *NPM1* mutation at diagnosis. This enabled comparison of *DNMT3A* stability during both CR and complete molecular remission (molCR) using the well-established marker for detection of MRD. The main characteristics, karyotype, concurrent mutations, percent of *DNMT3A* mutation, and MRD data of these patients are shown in Table 3. Median age was 55 years (range 43–60 years), and the median survival time by the end of this study was 30 months (range 9–52 months).

**Table 3:** Quantitative analysis of DNMT3A R882H in follow-up samples from 6 AML patients

Patient	Age/Gender	FAB	Karyotype	Mutations at diagnosis	Disease status	*DNMT3A* %	*NPM1* log	*FLT3* Ratio	Outcome/OS
1	52/F	M4	46,XX	*NPM1*	DS	21,45	1^10^6^	0,72	CR/9+
				*FLT3-*ITD	CR after CT	16,4	1^^-5^	0	
				*FLT3-*TKD2	**CR, day+28 after alloSCT , CDC**	**0**	**0**	**0**	
				*DNMT3A*	**CR, day+195 after alloSCT, CDC**	**0**	**0**	**0**	
2	60/M	M2	46,XY, del(9q)	*NPM1*	DS	11,95	1^10^5^		CR/31+
				*DNMT3A*	CR after IT	5,8	1^10^-2^		
					CR after CT	0,05	1^10^-4^		
					CR, Platelet ↓	0,1	1^10^-4^		
					CR	16,5	1^10^-5^		
					CR	30,8	0		
					CR	11,3	1^10^-4^		
					**CR, day+26 after alloSCT, 98 % DC**	**0**	**0**		
					**CR, day+73 after alloSCT (CDC)**	**0**	**0**		
3	43/F	M4	46,XX	*NPM1*	DS	17,9	1^10^5^	0,78	CR/52+
				*FLT3*-ITD	**CR, day+563 after alloSCT, CDC**	**0**	**1^10** ^**-5**^	**0**	
				*DNMT3A*	**CR, day+731 after alloSCT, CDC**	**0**	**1^10** ^**-4**^	**0**	
					**CR, day+1082 after alloSCT, CDC**	**0**	**0**	**0**	
					**CR, day+1445 after alloSCT, CDC**	**0**	**0**	**0**	
4	50/M	M4	46,XY	*NPM1*	DS	14,95	1,0^10^5^	0,41	R/17+
				*FLT3-*ITD	CR after IT	1,6	1^10^-4^	0	
				*DNMT3A*	CR after CT	1,2	1^10^-5^	0	
					**CR, day+40 after alloSCT, CDC)**	**0**	**1^10** ^**-4**^	**0**	
					**CR, day+130 after alloSCT, CDC**	**0,1**	**0**	**0**	
					**CR, day+217 after alloSCT, CDC**	**0**	**0**	**0**	
					**R, day+306 after alloSCT, 10 % DC**	**9,25**	**1^10** ^**5**^	**0**	
					**R, day+373 after alloSCT, 15 % DC**	**11,7**	**1^10** ^**4**^	**0,25**	
5	50/F	5a	46,XX	*NPM1*	DS	22,15	1^10^5^	0,5	CR/26
				*FLT3-*ITD	CR after IT	0,35	1^10^-4^	0	
				*DNMT3A*	CR after IT	0,2	1^10^-4^	0	
				*IDH1*	CR after CT	0,1	1^10^-4^	0	
					CR after CT	0,05	0	0	
					**CR, day+29 after alloSCT, CDC**	**0,05**	**1^10** ^**-4**^	**0**	
					**CR, day+71 after alloSCT, CDC**	**0**	**1^10** ^**-5**^	**0**	
					**R, day+114 after alloSCT, 90 % DC**	**0,05**	**1^10** ^**-4**^	**0**	
					**CR, day+174 after alloSCT, CDC**	**0**	**0**	**0**	
					**CR, day+215 after alloSCT, CDC**	**0**	**0**	**0**	
6	71/M	5a	46,XY	*NPM1*	DS	12,75	1^10^4^		CR/46+
				*DNMT3A*	CR after IT	0,1	1^10^-2^		
				*IDH1*	CR after CT	0,1	1^10^-4^		
				*IDH2*	CR after CT	1,65	1^10^-4^		
					CR	0,1	1^10^-4^		
					CR	4,05	1^10^-4^		
					CR	0,5	0		
					CR	0	0		
					CR	0	1^10^-4^		
					R	2	1^10^-2^		
					R	0,5	1^10^4^		
					2.CR	0	1^10^-4^		
					2.CR	0,05	1^10^-3^		
					**CR, day+27 after alloSCT, 92 % DC**	**0**	**1^10** ^**-4**^		
					**CR, day+80 after alloSCT, 60 % DC**	**0,45**	**0**		
					**CR, day+91 after alloSCT, 65 % DC**	**0,15**	**1^10** ^**-4**^		
					**CR, day+208 after alloSCT 14 % DC**	**1,25**	**0**		
					**CR, day+279 after alloSCT, CDC**	**0**	**0**		
					**CR, day+570 after alloSCT, CDC**	**0**	**0**		

*FAB* French–American–British classification systems, *DS* primary diagnosis, *IT* induction therapy, *CT* consolidation therapy, *CR* complete remission, *R* Relapse, *alloSCT* allogeneic stem cell transplantation, *CDC* complete donor chimerism, *DC* donor cells

+patient alive at the end of study. Samples after alloSCT are bold

For most analyzed patient, a *DNMT3A* R882H mutation was detectable after induction and consolidation therapy. The percentage of mutated allele was not constant and fluctuated in the course of disease. For example percentage of R882H in follow-up samples of patient 6 ranged from 0.05 % to 4.05 % during therapy. Furthermore, even in CR and molCR according to analysis of *NPM1*, a significant proportion of *DNMT3A* R882H mutation could be detected. In patients 1, 2, 3, 4, and 5 (Table 3), *DNMT3A* mutation was present at diagnosis and in CR after standard therapy. The amounts of mutation were decreased, for example, from 21.45 % to 16.5 % in patient 1 and from 14.95 % to 1.2 % in patient 4. In patient 2 (Table 3), amount of mutated allele was later increased despite the patients was in CR with slight positive or negative MRD. The percentage of mutated allele was higher (30.8 %) as compared to diagnostic sample (11.95 %). At this time point, the patient exhibited thrombocytopenia in peripheral blood with normal blasts count in BM. In samples obtained in CR after alloSCT (Patients 1, 2, and 3, Table 3) with complete donor chimerism (CDC), *DNMT3A* mutation was not found. Interestingly, *NPM1* mutation was found in very small amount. Overall, in CR no significant correlation between *NPM1* and *DNMT3A* was detected. *DNMT3A* mutation was not detectable in patients 1, 2, and 3 after alloSCT, in CR with CDC. After alloSCT no *DNMT3A* R882H mutation was detected in cases when the patient displayed a complete or high percent of donor cells.

In relapse of AML, significant proportion of *DNMT3A* R882H mutation was detected in all relapsed patients (Table 3). For instance, percentage of R882-positive allele increased significantly in patient 4 at relapse from 0 % to 9.25 % correlating with the molecular relapse detected by the percentage of *NPM1* and reduced amount of donor cells (10 %). In patient 6 the decreasing of donor cells from 92 % to 60 % associated with a growing of R882-positive allele from 0 % to 0.45 %. However, percentage of *NPM1* did not increase and a relapse was not diagnosed. This patient underwent match related alloSCT after reduced intensity conditioning, and demonstrated delayed engraftment with slowly growing of donor cells. Notably, in the CR, the majority of patients showed a high level of *DNMT3A* mutations despite the low or negative rates of *NPM1* mutation. In relapse, levels of both mutations have been increased.

## Discussion

Last years, potential role of *DNMT3A* mutation for prognosis and outcome of AML has been extensively studied. Several authors have shown a negative impact of *DNMT3A* mutations on outcomes of AML patient [12, 13, 15, 18, 27–29]. *DNMT3A* mutation has been shown an independent poor prognostic factor for overall survival and relapse-free survival [15]. Currently, only few methods for the quantitative detection of *DNMT3A* R882H mutations are available [19, 30]. Here, we developed an ASB-PCR assay that allows discrimination between wt and mutated *DNMT3A* and quantification of R882H allele in one reaction. The main feature of this assay is the combination of an allele-specific primer with a competitive blocker as described by Morlan *et al.* [25]. Features of *DNMT3A* R882H sequence allowed introduction of a purin-purin-mismatch (G-A) to the allele-specific primer leading to its high selectivity. Thus, a significant amplification difference for the wt and mutant allele was seen also without the use of the competitive blocker. Adding blocker enhanced selective amplification of R882H-mutated allele that lead to earlier detection of mutation. Furthermore, the maximum C_t_ difference between wt and mutated allele was 14 cycles. Thereby, effectiveness of the blocker is caused by intersection with the allele-specific primer and location of the discriminating base in the middle of the blocker sequence. In contrast, the allele-specific primer includes the discriminating base at its 3′-end.

In addition to the enabling of discrimination between wt and mutant allele, the assay had to facilitate analysis of follow-up samples that contain a low percentage of mutated allele. The high sensitivity of 10^−3^ and the possibility to use low DNA amounts, up to 1 ng, make this approach feasible.

Here, absolute quantification was performed by the percentage of mutated R882H allele in a sample. Since the generated plasmid contained a homozygous R882H mutation the amplification characteristics using ASB-PCR were different as compared to patient samples containing the heterozygous mutation. Possibly, the missing competition between wt and mutant allele during the amplification of plasmid DNA leads to a more efficient enrichment of mutant R882H generating higher C_t_ values. Modification of plasmid concentrations according to the percentage of mutated allele in the patient samples determined by sequencing enabled a reliable absolute quantification. Alternatively, the generation of an additional plasmid containing the wt allele is possible. Absolute quantification can be performed by combining the wt plasmid with different proportions of the plasmid containing the mutated allele [31].

For application of ASB-PCR in diagnostic routine the concordance with other analysis methods is important. ASB-PCR displayed a perfect concordance with sequencing and endonuclease restriction analysis [22]. This accounts for a high specificity of the assay. However, because low amplification of wt samples is present a wt control should be run together with patient samples. Here, the cut-off value of C_t_ = 35 was applicable in every run but this could depend on the PCR reagents and machine used for Real-Time quantification.

To demonstrate the need and effectiveness of new established method, we analyzed 62 follow-up samples from 6 AML patients after therapy and alloSCT. Moreover, data about the stability of *DNMT3A* mutation during the course of disease are restricted and controversial. Previously published studies have demonstrated that *DNMT3A* mutations are stable at relapse [12, 15]. Thol *et al.* found that the mutations disappeared at CR and recurred at relapse in one patient [12]. Later, Hou *et al.* reported persistence of *DNMT3A* mutations at CR in 5 patients, which later achieved relapse and died of disease progression. These data could relate the persistence of *DNMT3A* mutations and high risk of relapse [18]. Recently, Pløen *et al.* have identified persistence of *DNMT3A* mutations in long-term remission of patients with AML that received cytoreduction or palliative therapy [19]. Using cell-sorting, the authors showed that *DNMT3A mutations* were present in T-cells and B-cells at diagnosis in some patients, and also in T-cells several years after diagnosis. The presence of *DNMT3A* in both B-cells and T-cells could lead to the assumption that mutation had occurred in an early pre-leukemic stem cell prior to the acquisition of other genetic events, and could be resistant to chemotherapy [19]. Therefore, further exploration of the role of *DNMT3A* R882H mutations for the progression of AML disease is needed.

In accordance with previous studies, we found persistence of *DNMT3A* R882H mutations after standard cytoreduction therapy. In most cases the amount of mutated allele was lower as compared with the diagnostic sample. But in one case (Patient 2, Table 3) we have found an increasing of *DNMT3A* R882H allele in CR without relapse signs. Furthermore, *NPM1* remained negative indicating potential persistence of a different clone. The permanent presence of *DNMT3A* R882H after therapy in deep CR could be indicating presence of *DNMT3A* mutation in early pre-leukemic stem cells that resist chemotherapy. This could also explain the loss of correlation between *NPM1* and *DNMT3A* because the clonal expansion of pre-leukemic stem cells could originate independent of *NPM1* positive clones [10, 11]. In relapse, all samples showed an increasing of both *NPM1* and *DNMT3A* mutated alleles. This suggests at least in part the presence of *NPM1* and *DNMT3A* mutations in the same cell clone. After alloSCT in patients in CR with complete donor chimerism we have no found *DNMT3A* R882H. This data suggests the removal of leukemic stem cells after alloSCT and indicates the importance of alloSCT for high risk AML patients.

In summary, we developed a rapid, sensitive and specific method for quantitative detection of *DNMT3A* R882H mutations in AML patients. This assay could be easily applicable for routine screening of *DNMT3A* R882 mutation not only at time of diagnosis but also after the treatment. Quantitative detection of *DNMT3A* R882H mutations at different time points of AML disease enables screening of follow-up samples. This could assist to evaluate response to therapy and provide additional information about the role of *DNMT3A* mutations in development and progression of AML.
